# B-Virus and Free-Ranging Macaques, Puerto Rico

**DOI:** 10.3201/eid1003.030257

**Published:** 2004-03

**Authors:** Kristen Jensen, Francisco Alvarado-Ramy, Janis González-Martínez, Edmundo Kraiselburd, Johnny Rullán

**Affiliations:** * Cornell University, Ithaca, New York, USA; † Centers for Disease Control and Prevention, Atlanta, Georgia, USA; ‡ Puerto Rico Department of Health, San Juan, Puerto Rico; § Caribbean Primate Research Center, Sabana Seca, Puerto Rico

**Keywords:** macaques, rhesus, monkey, Cercopithecine herpesvirus 1, B-virus, Puerto Rico, exposure, zoonoses

## Abstract

In Puerto Rico, risk for transmission of B-virus from free-ranging rhesus monkeys to humans has become a serious challenge. An incident with an injured rhesus monkey, seropositive for B-virus, resulted in inappropriate administration of antiviral postexposure prophylaxis. This incident underscores the importance of education about risks associated with interactions between humans and nonhuman primates.

Two species of introduced nonhuman primates currently thrive on the Commonwealth of Puerto Rico: rhesus macaques (*Macaca mulatta*) and patas monkeys (*Erythrocebus patas*) (*1*). Although most of the monkeys live in groups in the southwest region of Puerto Rico, recent events might indicate that the primates have spread to the rest of the island, including urban areas.

Both species originated from the La Parguera Primate Facility, which was administered by the Caribbean Primate Research Center of the University of Puerto Rico’s Medical Sciences Campus from 1961 until 1982 (*2*). Primates were introduced onto two peninsulas, Isla Cueva and Isla Guayacan, off the southwest coast of Puerto Rico, near Guánica (Figure). In 1974, the center, through a contract with the Food and Drug Administration, began to increase the number of breeding female rhesus monkeys to supply animals for the Sabin Poliomyelitis Virus Vaccine Program. Throughout the 1970s, the rhesus colony numbers were increased to >1,000 (*3*,*4*). Patas monkeys were introduced to the peninsulas between 1971 and 1981. During this time, an unknown number of monkeys of both species escaped into the regions of Sierra Bermeja, Lajas, Cabo Rojo, and San German. La Parguera Primate Facility ceased operating in 1982, and the monkeys were removed from the facility. However, during the last 20 years, the escapees and their progeny have continued to cause problems in the area, plaguing farmers and concerning public health and environmental officials. Recently, an automobile in an urban area near San Juan (approximately 100 km from La Parguera) hit an adult rhesus monkey. During the incident, a number of emergency personnel were exposed to the monkey’s body fluids. The monkey subsequently tested positive for antibodies to B-virus (*Cercopithecine herpesvirus 1)*.

**Figure:**
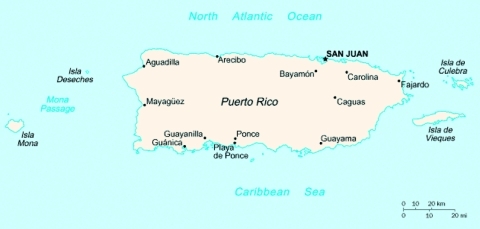
Map of the Commonwealth of Puerto Rico. Nonhuman primates were originally introduced in the southwestern coast, near Guánica.

## The Study

B-virus is an alphaherpesvirus enzootic among primates of the genus *Macaca* (e.g., rhesus, cynomolgus, pig-tailed). First documented in 1932, this virus has received considerable attention because rhesus macaques are a species commonly used in research. Data indicate that 74%–100% of adult rhesus monkeys are seropositive for the virus (*5*,*6*). In 1997, a survey of 57 rhesus monkeys in southwest Puerto Rico found that 41 (72%) had serologic evidence of B-virus infection (unpub. data).

Similar to herpes simplex infection in humans, B-virus might be asymptomatic or associated with mild lesions or conjunctivitis in macaques (*7*). Whereas the effects of the virus are mild in macaque hosts, it is a serious and frequently fatal disease in other primates, including humans. Once transmitted to a human, B-virus infection has a nearly 80% case-fatality rate. To date, <50 human infections have been reported in the literature since infection was first recognized in 1932, despite the multitude of exposures occurring primarily in laboratory environments (*7*,*8*). A survey of laboratory workers indicated asymptomatic seroconversion is unlikely (*9*). Most transmissions have occurred through monkey bites or scratches, but cases have been documented from needlestick injuries or other laboratory-related accidents, cage scratches, or mucous membrane exposure to monkey body fluids. One isolated case of human-to-human transmission was documented after a woman applied ointment to her husband’s infected wound on her ring finger, which had dermatitis (*10*). B-virus infection should be recognized early and antiviral prophylaxis given promptly. Early signs of infection in humans include influenzalike symptoms, such as headache, nausea, vomiting, and muscle pain. Vesicular herpetic lesions, pain, or itching might arise around the area of exposure. The disease progresses quickly to a fulminating meningoencephalitis. Symptoms include paralysis (often progressive and ascending), numbness, ataxia, respiratory difficulties, urinary retention, altered consciousness, and coma. The few cases that have been treated by the time neurologic symptoms have emerged have had limited success (*5*).

Like other herpesviruses, latency is a feature of B-virus, and many macaques will harbor the virus in trigeminal and lumbosacral ganglia. Stress will increase the likelihood that the virus will be reactivated and shed. Illness, transport, breeding, or environmental stresses have been reported as factors increasing a macaque’s likelihood of shedding the virus and therefore becoming infectious. Typically, a very low percentage (2%–3%) of seropositive monkeys will shed the virus at any given time (*5*). Risk for transmission, therefore, remains quite low.

In February 2002, a total of 25 emergency personnel were exposed to fluids from an injured adult male rhesus monkey that had been struck by an automobile in Bayamón, Puerto Rico. Most persons (n = 22) reported direct contact with the monkey’s blood, and none were bitten or scratched by the monkey. B-virus is not a bloodborne pathogen and is not transmitted by blood. However, the possibility of contact with other body fluids that represent a risk for B-virus transmission (e.g., saliva) could not be ruled out in four persons. All 25 persons were interviewed and evaluated by Puerto Rico’s Worker’s Compensation Agency and placed on prophylactic antiviral medication. All persons were employees from various municipal agencies responding to the accident. None wore latex gloves or other protective gear, despite the fact that most of the persons (84%) had access to personal protective equipment, including latex gloves, masks, and protective eyewear. Current protocols emphasize that the first few minutes after an injury or exposure are the most important (*7*). In this case, 1 person (4%) washed the exposed area within 1 to 2 minutes of exposure; 11 (44%) waited >5 minutes, 4 (16%) >30 minutes, and 9 (36%) waited >1 hour to clean the exposed area. Most persons used soap and water for cleansing; whether detergents or disinfectants were used is unknown. All 25 persons indicated that they were unaware of potential health risks involved in handling a primate or a rhesus monkey in particular. All were unaware of B-virus. None had been instructed to use protective equipment when handling a monkey. Subsequently, the rhesus monkey tested positive for antibodies to B-virus with enzyme-linked immunosorbent assays (ELISA). Paired serologic samples, completed at weeks 0 and 5 on all 25 persons, were negative for evidence of human infection by B-virus. The center’s Virology Laboratory performed the initial ELISA test for the blood sample from the monkey and also confirmed the positive result by sending aliquots of the sample to two independent laboratories (antibodies against B-virus were confirmed by Western Blot [B Virus Resource Laboratory, Atlanta, GA] and by ELISA [BioReliance Corporation, Rockville, MD]).

## Conclusions

For 70 years, considerable effort has been undertaken to understand the epidemiology of B-virus to decrease risk for human exposures in research laboratory settings. In the United States, several exposures have resulted from pet macaques (*8*); however most B-virus exposures occur inside the laboratory. Work continues to create specific pathogen-free rhesus colonies for research; in the meantime, laboratory personnel are kept well informed and every attempt is made to avoid transmission. Detailed protocols have been developed outlining recommended procedures for human exposure to macaques. Laboratory personnel depend on well-educated healthcare providers that can provide a prompt and knowledgeable management after potential exposures.

Puerto Rico health officials face a novel challenge: free-ranging rhesus monkeys in contact with a largely unaware public. Although the risk for B-virus transmission might be expected to be relatively low, as the monkeys continue to expand their range and population, further encounters with humans should be expected. Rough estimates suggest 500 macaques might be living in southwest Puerto Rico. Data indicate that rhesus populations typically increase their population by approximately 15% each year provided food is available (*11*).

In southwest Puerto Rico, crop predation is an ongoing problem. Farmers have implemented various tactics to protect their crops, including electric fences, dogs, and possibly hunting or trapping the macaques. Agricultural damage can be considerable after a group moves through a field. Reports document that persons are trapping monkeys, presumably for illegal sale as exotic pets. During a 1993 census, multiple traps were found set up in the forest (Janis González, unpub. data). This finding is alarming, not only from a legal but also from a public health perspective. Trapping and confinement could potentially stress an animal enough to initiate reactivation of latent virus. Inexperienced handlers are at increased risk for a bite or scratch, and fear of legal implications might prevent a person from reporting or seeking medical attention for such injuries. Healthcare providers inexperienced with primates and associated zoonotic diseases might be largely unaware or unfamiliar with B-virus.

Control of the free-ranging, nonhuman primate population promises to be a challenging task. Other examples of introduced primates in the Caribbean offer little consolation. The island of Desecheo is one example; it is located in the Mona Passage between Puerto Rico and Hispaniola (divided between Haiti and the Dominican Republic). In 1966, to study adaptation processes, a total of 57 rhesus macaques were released onto the remote 1.2-km^2^ island. The monkeys adapted well, and after nesting bird populations dropped radically on the island, government officials decided to remove the rhesus population (*12*). Thirty-six years later, after numerous trapping attempts, rhesus macaques still inhabit the island, a National Wildlife Refuge. Barbados suffers similar problems with an introduced species, the African green monkey (*Cercopithecus aethiops sabaeus*). This species was introduced >300 years ago with the slave trade. A study completed in 1994 indicated that despite trapping and removing more than 10,000 monkeys for 14 years, the population still increased by 4.5%; agricultural damage from the monkeys increased almost 30% during the same time span (*13*).

Although government agencies and other organizations continue to discuss interventions to address the free-ranging population of monkeys, the Puerto Rico Department of Health has begun the process of public education. Informational bulletins for government employees will stress the importance of using personal protective equipment when handling a nonhuman primate and what to do in case of an exposure to a rhesus monkey. In addition, every emergency room will receive a protocol for evaluation and management of exposed persons.

The persons exposed in the incident in Bayamón suffered a low-risk exposure. While the macaque was seropositive for B-virus, most the persons involved were only aware of exposure to blood; no bites or scratches occurred. Exposure to a macaque’s blood does not constitute an exposure to B-virus because viremia is thought to be rare among infected macaques (*14*). Nonetheless, 25 persons were needlessly exposed to fluids from a rhesus, postexposure cleaning was deficient for most persons, and all received an antiviral drug. Prevention of exposure and the unnecessary use of postexposure prophylaxis can be minimized through educational efforts to promote the use of personal protective equipment among first responders and through a better understanding of what body fluids constitute a risk for transmission of B-virus. Furthermore, the general public needs to be aware of this risk and report close contacts with monkeys to health authorities. Other emerging infections, such as monkeypox, underscore the need to remain vigilant against zoonoses.
